# The Story of the Insane from Year to Year

**Published:** 1894-09-01

**Authors:** 


### THE STORY OF THE INSANE FROM YEAR TO YEAR.

(Seventh Series.)

### GLOUCESTER COUNTY ASYLUMS.

The numbers fell in these asylums from 1,037 to 1,033, not, however, owing to a decrease in Gloucestershire cases, but to the discharge of some of the out-county patients. There were 279 admissions, 107 recoveries, and 100 deaths. The percentages of recoveries and deaths stand respectively at 41·31 and 9·63. Hereditary influence alone or in combination with other causes accounts for 33 of the admissions, and similarly drink is responsible for 24. Cerebral and spinal diseases caused death in 39 cases, and consumption in 7. Fourteen private patients were admitted during the year, and Dr. Craddock has some well-expressed remarks about the presence of this class in county asylums. He says: "It would be desirable if some scheme could be devised for keeping them in wards of their own, apart from the ordinary pauper patients. They do not mix well; the private patients are many of them dissatisfied themselves, and a cause of discontent among the paupers with whom they associate." And Dr. Craddock adds that the solution of the difficulty lies in providing detached accommodation for private patients. We are satisfied this is the right view to take, as such provision would be advantageous to the pauper part of the asylum, a great blessing to the middle class lunatic, and a source of profit to the county. There were two cases of typhoid fever, and here again neither the medical officer of health nor the medical superintendent was able to assign an adequate cause for the outbreak. The visitors say that "under the able management of Mr. Craddock the asylums have been maintained in good order and to the entire satisfaction of the committee." The weekly charge is 8s. 2d.

### THE RICHMOND ASYLUM, DUBLIN.

Here the limits of accommodation are stated to be for 1,100 patients, and yet the asylum contained on January 1st, 1893, 1,467, which number was increased to 1,498 by December 31st. There were 499 admissions, 163 recoveries, and 187 deaths. The recoveries reached 32·6 per cent., and the deaths were 12·5. Hereditary influence is the assigned cause of insanity in 152 of the admissions, and drink in 106, showing 50 per cent. of the admissions had no right to exist at all; and although this state of things has been pointed out over and over again, we are still endeavouring to discover "cures" for insanity, instead of carrying out preventive measures. Cerebral and spinal diseases caused death in 24 cases, and among these we note that 13 were from general paralysis, while consumption was the cause in not fewer than 82 cases; and this number, high as it is, does not include 8 deaths from other tubercular diseases. There can hardly be a doubt that the crowded state of the asylum had something to do with this extensive mortality, and Dr. Norman points out the "paramount importance of abundance of air and space, and the ruinous results of living in a contaminated atmosphere." The weekly cost of maintenance is 9s. 1½d.

### THE JOINT COUNTIES' ASYLUM AT ABERGAVENNY.

This is the asylum for Monmouth, Brecon, and Radnor. It contained on January 1st 917 patients, and by December 31st the number had risen to 935. There were 230 admissions, 76 recoveries, and 77 deaths. The recoveries stand at 33 per cent. of the admissions, and the deaths at 8·2 on the average number resident. Of the year's admissions 39 were due to hereditary tendency and 20 to intemperance in drink. Cerebral and spinal diseases caused death in 36 cases and consumption in 9. The in-county patients increased by 54, and Dr. Glendinning does not attribute this increase altogether to an actual increase of insanity in the district; but "is no doubt in part owing to the better treatment received in the asylums, and the greater confidence manifested by the outside public in the management of asylums; but in greater part to the county grant of 4s. per week towards the cost of maintenance of each patient in the asylum, and to our low weekly charge of 7s. 7d., leaving the cost to the unions only 3s. 7d., or less than the patient can be kept for in the workhouse or at his own home." The wisdom of that 4s. grant has never yet been demonstrated, and its drawbacks have been pointed out many a time. The committee say they "have pleasure in stating that, under the able management of the medical superintendent, the efficiency and comfort of the establishment have been fully maintained."

### NORTHAMPTON COUNTY ASYLUM.

On January 1st there were in this asylum 863 patients, and on the last day of the year the number was 859. The admissions were 197, the recoveries 43, and the deaths 67. The percentage of recoveries was 25·4, and the deaths 7·7. Hereditary taint was the cause of the insanity in 63 of the admissions, and drink in 10. Brain disease is responsible for 37 of the deaths, and consumption for 5. The number of registered lunatics seems to be increasing rapidly in Northamptonshire, for we notice that the asylum contained 44 more in-county patients than it did at the beginning of the year. The training of attendants has made considerable strides in the female department of the asylum. "The system pursued," says Mr. Greene, "has been to divide the training into three courses of one year each, the first course consisting chiefly of surgical work; the second, chiefly of medical nursing; and the final year to the management of mental cases. A broad distinction is drawn between mere residence and training, and the former is not reckoned unless the latter run with it." The training and certificates thus closely resemble those given in hospitals, and we notice that 14 nursing institutions have signified their intention of accepting the Berry Wood certificate as qualifying for admission to membership. There has been a scarcity of water during the year, but a tender for sinking a new well has been accepted. The weekly charge to the unions is 7s. 6d. Out-county patients pay 14s.

### KIRKLANDS ASYLUM, GLASGOW.

This is a small district asylum, containing on the last day of the year 228 patients, and it only increased its numbers by one, which is a record rare indeed in other similar institutions. There were 89 admissions; 33 recoveries and 24 deaths. These numbers yield a percentage of 37 recoveries and of 7·6 deaths. Drink heads the list of causes of insanity, and accounts for 18 of the year's admissions, while hereditary influence is only responsible for five. Cerebral and spinal diseases were the causes of death in 12 cases, and consumption in one case. Dr. Campbell Clarke's report is an interesting and eminently readable one. He rapidly runs over a great many topics connected with asylums and insanity. He tells us he has had twenty years of asylum work, and he has seen much progress in asylums and in the care of the insane. The old "keeper" has been changed into an "attendant" or "nurse," and the insane are now patients and not prisoners. If Dr. Clarke will study the new Lunacy Act he will find that the "prisoner" idea is not wholly banished from English egislation; and if he will cross the Tweed and visit a few English asylums he will too surely find airing-court walls in full force and locked doors all but universal. We move slowly in England, but, perhaps, safely, and we have done something to justify Dr. Clarke's remark that "when theory comes down from the clouds we get practice, and now we have hospitals for the insane, not on paper, but in stone and lime, properly heated, well ventilated, comfortably furnished, medically equipped up to date, and with a liberal dietary"; and further on he says, "Tradition and popular ignorance too often associate asylum attendants and nurses with everything that is hard-hearted, unfeeling, and unkind; but the public ought to know that the majority of our asylums are getting thoroughly purged of the old keeper system, just as our hospitals are now purged of their Mother Gamps." For our own part we believe that our best county asylums are in this particular not only abreast of hospitals but considerably ahead of them; and their staffs would include a proportion of hard-working, conscientious, and even-tempered men and women not to be found in other institutions. When speaking of the destructive habits of the insane, Dr. Clarke thinks the question of cost of the article destroyed passes through the mind of the lunatic oftener than is supposed; but he hints it may be a characteristic of the Scotch lunatic. It certainly is not of an English one. He destroys indiscriminately. The weekly rate of maintenance is 8s. 9d. per head.

